# Ganglioside GM3-Functionalized Reconstituted High-Density Lipoprotein (GM3-rHDL) as a Novel Nanocarrier Enhances Antiatherosclerotic Efficacy of Statins in apoE^−/−^ C57BL/6 Mice

**DOI:** 10.3390/pharmaceutics14112534

**Published:** 2022-11-20

**Authors:** Bo Wei, Yuanfang Li, Meiying Ao, Wenxiang Shao, Kun Wang, Tong Rong, Yun Zhou, Yong Chen

**Affiliations:** 1 College of Life Sciences, Nanchang University, Nanchang 330031, China; 2 Jiangxi Key Laboratory for Microscale Interdisciplinary Study, Institute for Advanced Study, Nanchang University, Nanchang 330031, China; 3 School of Chinese Medicine & Life Science, Jiangxi University of Chinese Medicine, Nanchang 330025, China

**Keywords:** ganglioside GM3, atherosclerosis, reconstituted high-density lipoprotein (rHDL), lovastatin (LT), drug delivery system, statins, ApoA-I

## Abstract

Previously, we found that exogenous ganglioside GM3 had an antiatherosclerotic efficacy and that its antiatherosclerotic efficacy could be enhanced by reconstituted high-density lipoprotein (rHDL). In this study, we hypothesized that GM3-functionalized rHDL (i.e., GM3-rHDL) as a nanocarrier can promote the efficacy of traditional antiatherosclerotic drugs (e.g., statins). To test this hypothesis, lovastatin (LT) was used as a representative of statins, and LT-loaded GM3-rHDL nanoparticle (LT-GM3-rHDL or LT@GM3-rHDL; a mean size of ~142 nm) and multiple controls (e.g., GM3-rHDL without LT, LT-loaded rHDL or LT-rHDL, and other nanoparticles) were prepared. By using two different microsphere-based methods, the presences of apolipoprotein A-I (apoA-I) and/or GM3 in nanoparticles and the apoA-I-mediated macrophage-targeting ability of apoA-I/rHDL-containing nanoparticles were verified in vitro. Moreover, LT-GM3-rHDL nanoparticle had a slowly sustained LT release in vitro and the strongest inhibitory effect on the foam cell formation of macrophages (a key event of atherogenesis). After single administration of rHDL-based nanoparticles, a higher LT concentration was detected shortly in the atherosclerotic plaques of apoE^−/−^ mice than non-rHDL-based nanoparticles, suggesting the in vivo plaque-targeting ability of apoA-I/rHDL-containing nanoparticles. Finally, among all nanoparticles LT-GM3-rHDL induced the largest decreases in the contents of blood lipids and in the areas of atherosclerotic plaques at various aortic locations in apoE^−/−^ mice fed a high-fat diet for 12 weeks, supporting that LT-GM3-rHDL has the best in vivo antiatherosclerotic efficacy among the tested nanoparticles. Our data imply that GM3-functionalized rHDL (i.e., GM3-rHDL) can be utilized as a novel nanocarrier to enhance the efficacy of traditional antiatherosclerotic drugs (e.g., statins).

## 1. Background

Gangliosides are a large family of glycosphingolipids (GSL) containing one or more sialic acid residues. As the simplest member of the ganglioside family, ganglioside GM3 contains only one sialic acid residue and is the precursor of all complex gangliosides [1]. Based on diversity of ceramide structures which are composed of sphingosine and fatty acids, several dozen GM3 molecular species exist due to different combinations of chain length, hydroxylation, and unsaturation of fatty acid chains [2]. GM3 has been reported to play important roles in many cellular activities (e.g., cell recognition, adhesion, and signal pathways) and to corelate with some diseases including cancers [3,4], impaired hearing [5,6], wound healing [7,8], diabetes [9,10], arthritis [11,12], as well as atherosclerosis [13,14]. Accumulating evidence supports that exogenous GM3 and its mimetics can be used to treat these diseases [15,16]. Recently, via both in vitro and in vivo experiments, we also reported that exogenous GM3 has an inhibitory efficacy against atherosclerosis by influencing multiple atherosclerotic steps (e.g., the size, charge, stability, oxidation susceptibility, cellular recognition/internalization of low-density lipoproteins (LDL), monocyte-adhering ability of endothelial cells, and lipid deposition in macrophages), implying its potential of being recruited as an antiatherosclerotic drug [13].

High-density lipoprotein (HDL) is one of the five major plasma lipoproteins that are the nanocarriers of lipids in blood circulation. Naturally, HDL functions as a cholesterol transporter during the reverse cholesterol transport (RCT) from the peripheral tissues to the liver for cholesterol excretion. Similar to other lipoprotein types, HDL also has a core–shell structure with an esterified cholesterol-containing hydrophobic core surrounded by a monolayer shell which is mainly made up of phospholipids, free cholesterol, and apolipoproteins (e.g., apoA-I and apoA-II). Due to this specific core–shell structure, other molecules (particularly lipophilic molecules) can also be delivered by HDL. Inspired by this structural property, reconstituted HDL (rHDL) has been developed as a novel drug delivery system and applied to transport different drugs for various diseases [17,18].

Another specific property of HDL/rHDL is the existence of apoA-I capable of being specifically recognized by SR-BI (i.e., scavenger receptor class B type 1, a HDL receptor) [19,20], which is highly expressed on the surfaces of macrophages in atherosclerotic lesion and tumors [21,22,23]. Therefore, rHDL nanoparticles have the ability of targeting atherosclerotic plaques and have been applied as a drug nanocarrier in the treatment of atherosclerosis [24,25,26,27]. Recently, we also tested and found that exogenous GM3 delivered by rHDL (i.e., GM3-rHDL) has a better antiatherosclerotic efficacy than exogenous GM3 alone due to the lesion-targetability of rHDL [28]. Therefore, exogenous GM3 can be regarded as an antiatherosclerotic drug which is delivered by rHDL. On the other hand, GM3 is a glycosphingolipid which can integrate into the phospholipid monolayer of rHDL. From this perspective, the GM3-rHDL nanoparticle can be regarded as a novel nanocarrier with enhanced antiatherosclerotic efficacy. Then, we hypothesized that the GM3-rHDL nanoparticle as an upgraded delivery system of rHDL can deliver other traditional antiatherosclerotic drugs (e.g., statins) for further improvement of the antiatherosclerotic efficacy. In this study, lovastatin (LT) was utilized as a traditional antiatherosclerotic drug to test the hypothesis.

## 2. Materials and Methods

### 2.1. Preparations of Nanoparticles

Nanostructured lipid carriers (NLC), reconstituted high-density lipoprotein (rHDL), GM3-rHDL, and LT-loaded nanoparticles were prepared by the thin-film dispersion method. Briefly, 45 mg egg phospholipid (PC, Lipoid E80; Lipoid GmbH, Germany), 10 mg cholesterol (Solarbio Science & Technology Co., Shanghai, China), 20 mg cholesteryl oleate, 15 mg glycerol trioleate, and 5 mg octadecylamine (the latter three were from Sigma, Saint Louis, MO, USA) were dissolved in 15 mL of methanol/chloroform (1:1, *v/v*) with or without 5 mg lovastatin (LT; Aladdin, Shanghai, China). Then, the lipid mixture was mixed with or without 0.5 mg ganglioside GM3 (sodium salt; AdipoGen, Fuellinsdorf, Switzerland) in an egg-plant flask and dried by a rotary evaporator (RE2000A; Shanghai Yarong Biochemistry Instrument Factory, Shanghai, China) at 60 rpm under vacuum at 45 °C for 1 h to remove the organic solvent. After adding 15 mL of 0.02 M Tris buffer (pH 8.0) containing 10 mg sodium cholate (Solarbio, Shanghai, China) in the flask and rotating again at 60 rpm at 45 °C for 1 h, the thin film was dispersed by vortexing for 15 min and ultrasonicating in an ice bath. After filtering with a 0.22 μm sterile filter, the NLC, GM3-NLC, LT-NLC, and LT-GM3-NLC suspensions were obtained, respectively. Next, 50 μg of human recombinant apoA-I (Cloud-clone, Katy, TX, USA) was added into 2 mL of LT-NLC or GM3-NLC or LT-GM3-NLC and shook at 100 rpm at 37 °C for 2 days. After removing free LT and sodium cholate by dialyzing in a 10 kDa dialysis bag (Solarbio, Shanghai, China) at 4 °C for 2 days, LT-rHDL (or LT@rHDL), GM3-rHDL, and LT-GM3-rHDL (or LT@GM3-rHDL) suspensions were obtained, respectively, and used immediately or stored at 4 °C.

### 2.2. Validation of apoA-I and/or GM3 in Prepared Nanoparticles via a Microsphere-Based Method

A microsphere-based method was used to determine the presences of apoA-I and GM3 in apoA-I-/GM3-containing nanoparticles, as reported in our previous studies, with minor revision [28]. Briefly, the commercial streptavidin-coated silica microspheres/beads (Bangs Laboratories, USA) were diluted to 1 × 105 beads/mL in biotin–streptavidin binding buffer, washed three times with PBS to remove the reagents (e.g., EDTA) in a commercial product, and resuspended in 1 mL PBS. The streptavidin-coated beads were incubated successively with biotinylated anti-apoA-I antibody (Cloud-Clone, Katy, TX, USA) at 37 °C for 1 h and with the nanoparticle suspensions at 37 °C for 12 h. After washing with PBS, the beads were resuspended in PBS for the following experiments. For the determination of apoA-I presence in nanoparticles, the streptavidin-coated beads were then stained with Nile Red (10 μg/mL; Sigma, Burlington, MA, USA) at 37 °C for 20 min in dark. After washing three times with PBS, the beads were subjected to confocal microscopy (Carl Zeiss, Oberkochen, Germany) and flow cytometry (Thermo, Waltham, MA, USA), respectively. For the determination of GM3 presence in nanoparticles, the streptavidin-coated beads were further incubated successively with anti-GM3 IgM (Amsbio, Abingdon, UK) at 37 °C for 1 h and with AlexaFluor488-conjugated goat anti-mouse IgM antibody (Invitrogen, Carlsbad, CA, USA) at 37 °C for 1 h. After washing with PBS, the beads were subjected to confocal microscopy and flow cytometry, respectively.

### 2.3. Characterizations of the Main Nanoparticles

The mean size, polydispersity index (PDI), and zeta potential of LT-NLC, LT-rHDL, GM3-rHDL, and LT-GM3-rHDL nanoparticles were measured by dynamic light scattering (DLS) Analyzer (Zetasizer nano zs90, Malvern, UK) as previously reported. The morphologies of LT-NLC, LT-rHDL, GM3-rHDL, and LT-GM3-rHDL nanoparticles were detected by a transmission electron microscope (JEOL JEM-2100 TEM, Japan) after staining with 2% (*w/v*) uranyl acetate.

The concentration of LT-loaded in nanoparticles was measured by the HPLC method. A COSMOSIL 5C18-MSII column (250 mm × 4.6 mm) was used at 30 °C. Approximately 80% methanol (*v/v*) of chromatographic grade was utilized as the mobile phase (flow rate: 1 mL/min). The detected wavelength was 238 nm. The standard curve was achieved by using standard LT solutions (1, 2, 4, 8, and 16 μg/mL). The entrapment efficiency (EE) and drug loading efficiency (DL) of LT-loading nanoparticles were calculated according to the following equations: EE (%) = W/W_t_ × 100% and DL (%) = Q/Q_t_ × 100%, where W and Q are the amount of LT in each drug carrier whereas W_t_ and Q_t_ are the total amount of the feeding LT and the feeding materials.

### 2.4. In Vitro Determination of the Macrophage-Targeting Ability of apoA-I-Bearing Nanoparticles via Another Microsphere-Based Method

Mouse RAW264.7 macrophages were purchased from Xiangya Central Experiment Laboratory (Xiangya, China) and routinely cultured in RPMI 1640 medium (Gibco, New York, NY, USA) supplemented with 10% FBS and 1% antibiotic mixture (100 Unit/mL penicillin and 100 μg/mL streptomycin). The cells were used at passage ~5 in the experiments.

Another microsphere-based method was utilized to determine the macrophage-targeting ability of apoA-bearing nanoparticles as reported in our previous studies with minor revision [28]. Briefly, the commercial streptavidin-coated silica beads were diluted to 1 × 10^5^ beads/mL in biotin-streptavidin binding buffer, washed three times with PBS, and resuspended in 1 mL PBS. The streptavidin-coated beads were incubated in a 5% CO_2_ incubator successively with biotinylated anti-apoA-I antibody (or with the buffer as a control) at 37 °C for 1 h and with the nanoparticle suspensions at 37 °C for 12 h. After washing with PBS and resuspending in PBS, the beads were incubated with RAW264.7 macrophages in a petri dish at 37 °C for 1 h. After rinsing three times with PBS to remove free beads, the cells were fixed with 2.5% glutaraldehyde for 20 min and subjected to an inverted microscope (Nikon LH-M100CB, Japan). The number of beads on each cell and the percentage of bead-bearing cells in total cells were calculated for quantitative analysis.

### 2.5. In Vitro Determination of the Inhibitory Effect of Nanoparticles on ox-LDL-Induced Lipid Deposition in Macrophages

Oxidized low-density lipoprotein (oxLDL) was used to develop an atherosclerotic cell model by stimulating lipid deposition in macrophages (or foam cell formation). In this experiment, the cells with no oxLDL stimulation and no nanoparticle treatment and the cells with oxLDL (100 μg/mL) stimulation but no nanoparticle treatment were recruited as a blank control group and a model group, respectively, whereas in the other groups the cells were stimulated/treated by both oxLDL (100 μg/mL) and nanoparticles. After incubating in a 5% CO_2_ incubator at 37 °C for 24 h, the cells were washed three times with PBS, fixed with 2.5% glutaraldehyde for 20 min, washed three times again with PBS, and stained with Oil Red O by treating cells successively with 60% isopropanol for 3 min, Oil Red O (Solarbio, Beijing, China) for 20 min, and 60% isopropanol three times each for 1 min. After washing with PBS, the cells were observed by the inverted microscope, and the lipid deposition in cells (i.e., the ratio of the red area in cells to the total area of cells in an image) was analyzed by ImageJ software.

### 2.6. Animals, Diet, and Treatments

Eight-week-old male apoE^−/−^ C57BL/6 mice (~21–23 g) were purchased from Beijing Vital River Laboratory Animal Technology Co., Ltd. (Beijing, China). To establish atherosclerotic mouse model, the mice were fed a high-fat diet (Hunan SJA Lab Animal Ltd., Changsha, China) which contains 21% fat, 0.15% cholesterol, and basic forage. Ethics approval for this study was obtained from the Ethics Committee of Jiangxi University of Chinese Medicine (Approval number: JZLLSC2017-205; 28 December 2017). All animal experiments were performed in full compliance with the National Institute of Health Guide for the Care and Use of Laboratory Animals.

For the in vivo experiments of drug efficacy, the apoE^−/−^ mice were randomly divided into the following 6 groups (n = 6 in each group): a control group (mice fed a chow diet for 12 weeks), a model group (mice fed a high-fat diet for 12 weeks), and the groups fed a high-fat diet for 12 weeks and meanwhile intravenously treated once every three days with ~200 μL of each of the nanoparticle solutions (for the control and model groups, ~200 μL of saline was administrated; the concentration of GM3 was 0.3 mg/kg for GM3 or GM3-containing groups) via tail vein injection. For the in vivo experiments of drug pharmacokinetics and tissue distribution, apoE^−/−^ mice were also fed a high-fat diet. After 12 weeks, the mice were fasted for 12 h and subjected to the following experiments.

### 2.7. Lipid Profiling of Blood Samples

At the end of the 12-week treatments of nanoparticles, the mice were fasted for 8 h, and the blood of each mouse was collected for serum preparation. The concentrations of four major lipids in sera including total cholesterol (TC), triglyceride (TG), low-density lipoprotein-cholesterol (LDL-C), and high-density lipoprotein-cholesterol (HDL-C) were measured with an automatic biochemical analyzer (Beckman Coulter AU480, Brea, CA, USA) by using corresponding Kits (Anhui Iprocom Biotechnology Co., Ltd.; Hefei, China).

### 2.8. Detection of Atherosclerotic Lesions in Full-Length Aorta, Aortic Arches, and Aortic Roots

Atherosclerotic lesions in different parts of aorta of each mouse were processed, imaged, and quantified as reported previously [28]. For the atherosclerotic lesions in aortic arch, the heart and aortic arch on a blue background were photographed immediately after perfusion. Then, the heart coupling with entire aorta including the aortic arch, thoracic aorta, and abdominal aorta was taken from each mouse, and cut into two pieces, i.e., the aortic part for imaging the atherosclerotic lesions in full-length aorta and the heart part for imaging the atherosclerotic lesions in aortic root. The aortic part was fixed with 4% paraformaldehyde, opened longitudinally, stained with Oil Red O, and imaged. The heart part was dehydrated in 30% sucrose solution at 4 °C for >12 h, frozen rapidly, embedded in tissue OCT-freeze medium, sliced (a thickness of ~8–10 μm), fixed with 95% ethanol, stained with hematoxylin-Oil Red, and imaged.

### 2.9. Measurements of In Vitro Drug Release and of In Vivo Drug Pharmacokinetics and Drug Distribution in Different Tissues

The in vitro drug release profile of LT from LT-loaded nanoparticles (LT-NLC, LT-rHDL, and LT-GM3-rHDL, respectively) was detected by using the dialysis method and using a dialysis bag with a molecular weight cut-off of ~10 kDa, as previously reported [28]. The dialysis of each sample (5 mL in a dialysis bag) was performed in 200 mL of release buffer (0.05% SDS in PBS, pH 7.4) at 25 °C for 72 h which was stirred at 50 rpm. At each indicated time point, 0.5 mL of release buffer was taken out (replaced by 0.5 mL of fresh buffer) and detected via HPLC to determine the released LT concentration.

For the evaluation of in vivo drug pharmacokinetics, the concentrations of LT in blood were measured at different time points (5 min, 15 min, 30 min, 1 h, 2 h, 3 h, 4 h, 6 h, 8 h, 12 h, 24 h, 36 h, and 48 h, respectively) after a single drug administration of the LT-loading nanoparticles (i.e., LT-NLC, LT-rHDL, and LT-GM3-rHDL, respectively; 2 mg/kg of LT), as previously reported [28]. Briefly, an aliquot of 200 μL plasma samples were mixed with 1 mL methanol of chromatographical grade and 3 μg simvastatin (internal standard), centrifugated at 12,000 rpm for 5 min, filtered with 0.22 μm filter, and subjected to HPLC for LT concentration measurements. A standard curve was achieved by using standard LT solutions (1, 2, 4, 8, and 16 μg/mL) in each of which 2 μg simvastatin was added as an internal standard.

For evaluation of the in vivo distribution of LT in different tissues (the blood, heart, liver, lung, spleen, kidney, and atherosclerotic lesions, respectively), the concentrations of LT in these tissues were measured at 0.5 h after a single drug administration (i.e., LT-NLC, LT-rHDL, and LT-GM3-rHDL, respectively; 2 mg/kg of LT), as previously reported [28]. Briefly, after being taken, weighted, and cut into small pieces, approximately 0.2 g tissues were mixed with 2 μg simvastatin (internal standard) and homogenized thoroughly in 1 mL of methanol. After centrifugation at 12,000 rpm for 10 min and filtration via a filter of 0.22 μm, the samples were subjected to HPLC. The LT standard curve was also achieved by using standard LT solutions with simvastatin as the internal standard.

### 2.10. Statistical Analysis

All data from at least three independent experiments are expressed as the mean ± SD. Student’s *t*-test between two groups or one-way ANOVA among multiple groups was used for statistical analyses. When *p* < 0.05, a statistically significant difference was considered. For post hoc analysis during one-way ANOVA, Bonferroni’s multiple comparisons test was performed.

## 3. Results and Discussion

To test our hypothesis, the traditional antiatherosclerotic (also cholesterol-lowering) drug lovastatin was encapsulated in GM3-rHDL nanoparticles producing LT-GM3-rHDL (or LT@GM3-rHDL) nanoparticles. For purpose of comparison, LT-loaded nanostructured lipid carrier (i.e., LT-NLC or LT@NLC), LT-loaded rHDL (i.e., LT-rHDL or LT@rHDL), and GM3-rHDL nanoparticles were also prepared.

**Figure 1:**
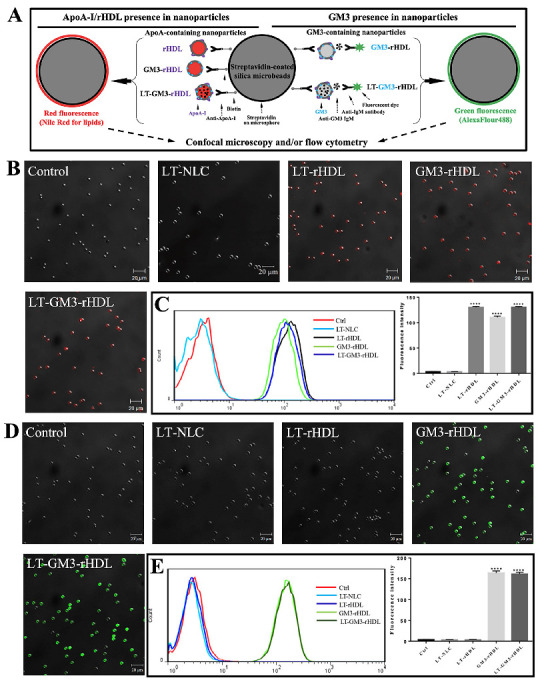
Validation of ApoA-I and/or GM3 in LT-rHDL (or LT@rHDL), GM3-rHDL, and LT-GM3-rHDL (or LT@GM3-rHDL) nanoparticles via a microsphere-based method. (**A**) Schematic diagram showing the principle of the method. (**B**,**C**) Validation of apoA-I via confocal microscopy (**B**) and flow cytometry (**C**), respectively. Streptavidin-coated silica beads were first coated with biotinylated anti-apoA-I antibody, then incubated with different samples (PBS or control, LT-NLC or LT@NLC, LT-rHDL or LT@rHDL, GM3-rHDL, LT-GM3-rHDL or LT@GM3-rHDL, respectively), and finally stained with Nile Red O (red). (**D**,**E**) Validation of GM3 via confocal microscopy (**D**) and flow cytometry (**E**), respectively. Streptavidin-coated silica beads were first coated with biotinylated anti-apoA-I antibody, then incubated with different samples, subsequently labeled with anti-GM3 antibody, and finally stained with AlexaFluor488-conjugated secondary antibody (green). (**C**,**E**) Left: representative flow cytometric data; right: quantification of mean fluorescence intensity of beads (**** *p* < 0.0001 compared with the control).

Prior to the characterization of these nanoparticles, the presences of apoA-I and/or GM3 in the nanoparticles were verified by using a microsphere-based method. For the validation of apoA-I in apoA-I-containing nanoparticles (e.g., LT-rHDL, GM3-rHDL, and LT-GM3-rHDL nanoparticles), nanoparticles were conjugated with streptavidin-coated silica beads (~5 μm in diameter) by using biotinylated anti-apoA-I antibody as a linker and fluorescently stained with Nile Red O in red for the lipids in the nanoparticles (left in Figure 1A); for the validation of GM3 in GM3-containing rHDL nanoparticles (e.g., GM3-rHDL and LT-GM3-rHDL nanoparticles), nanoparticles were conjugated with streptavidin-coated silica beads by using biotinylated anti-apoA-I antibody as a linker and fluorescently stained by incubating first with anti-GM3 antibody and then with AlexaFluor488-conjugated secondary antibody in green (right in Figure 1A); the successful detection of fluorescence on beads could reflect the existence of apoA-I or GM3 in nanoparticles (Figure 1A). The experimental data show that red fluorescence was detected on the beads in each of the rHDL, GM3-rHDL, and LT-GM3-rHDL groups (Figure 1B,C) and green fluorescence was observed on the beads in each of the GM3-rHDL and LT-GM3-rHDL groups (Figure 1D,E) by confocal microscopy and flow cytometry, respectively. The results confirmed that rHDL, GM3-rHDL, and LT-GM3-rHDL nanoparticles contained apoA-I molecules and that GM3-rHDL and LT-GM3-rHDL nanoparticles contained GM3 molecules.

**Figure 2:**
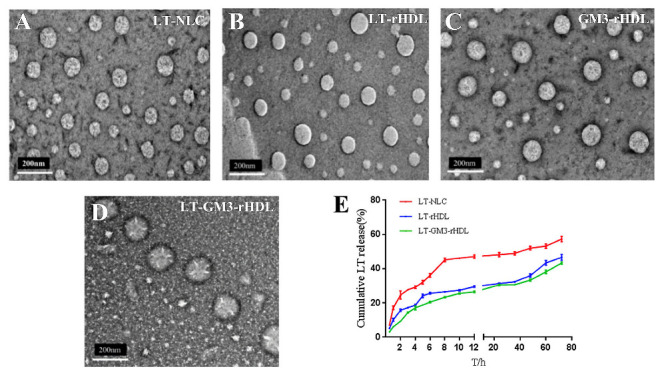
Transmission electron microscopic (TEM) images of LT-NLC or LT@NLC (**A**), LT-rHDL or LT@rHDL (**B**), GM3-rHDL (**C**), and LT-GM3-rHDL or LT@GM3-rHDL (**D**) nanoparticles, respectively, and (**E**) in vitro drug (LT) release profiles from LT-NLC, LT-rHDL, and LT-GM3-rHDL nanoparticles, respectively.

Then, the morphology and size of these nanoparticles were characterized by transmission electron microscopy (TEM) and dynamic light scattering (DLS) analyzer. All the nanoparticles displayed a spherical shape and a size of less than 200 nm in diameter (Figure 2A–D), and the DLS analyzer quantified a slightly larger mean size (~142 nm) of the LT-GM3-rHDL nanoparticle than that (~128 nm and ~115 nm, respectively) of the LT-rHDL or GM3-rHDL nanoparticle (Table 1). The entrapment efficiency (EE) and drug loading efficiency (DL) of lovastatin in the LT-GM3-rHDL nanoparticle were ~74.7% and ~4.75%, respectively (other LT-loaded nanoparticles had similar EE and DL values; Table 1). The in vitro drug release profiling revealed that both the LT-rHDL and LT-GM3-rHDL nanoparticles have a much more slowly sustained release of lovastatin than the LT-NLC nanoparticle (Figure 2E).

**Figure 3:**
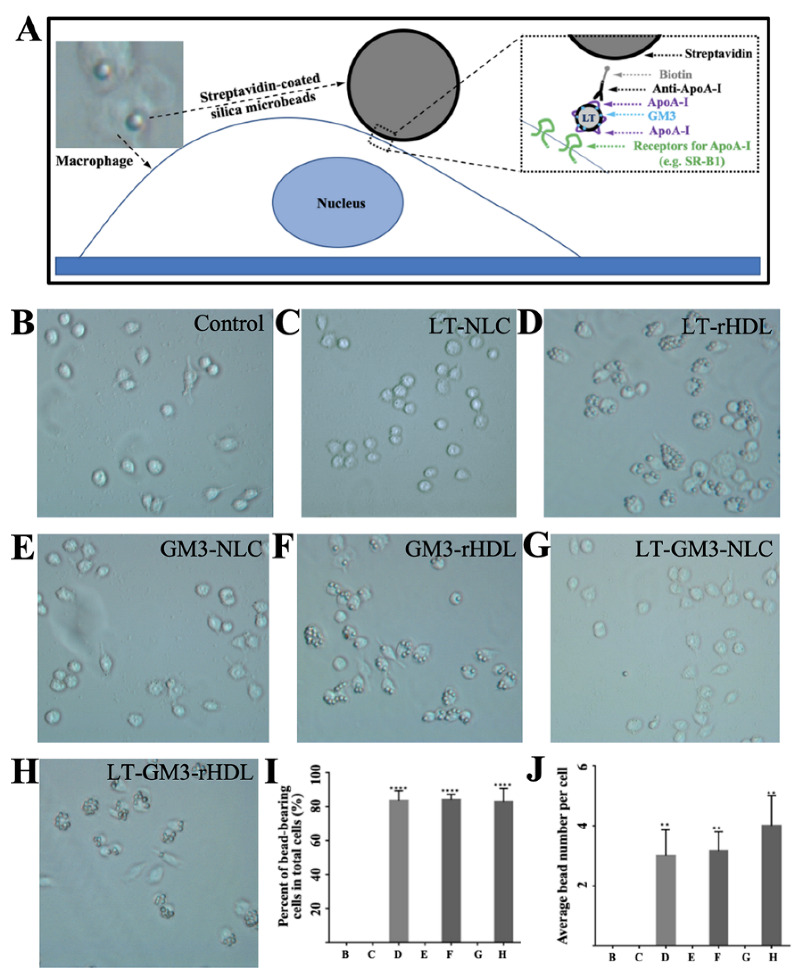
In vitro validation of cell-targeting ability of apoA-I-bearing (i.e., rHDL) nanoparticles via another microsphere-based method. (**A**) Schematic diagram showing the basic principle of the method. Streptavidin-coated silica beads were first coated with biotinylated anti-apoA-I antibody, then incubated with different samples (PBS or control, LT-NLC, LT-rHDL, GM3-NLC, GM3-rHDL, LT-GM3-NLC, LT-GM3-rHDL, respectively), and finally incubated with RAW264.7 macrophages expressing scavenger receptors (SR-B1) which can specifically recognize apoA-I. (**B**–**H**) Representative image in each group (magnification: 200×). (**I**) Quantification of the percentage of bead-bearing cells in total cells. (**J**) Quantification of the average bead number per cell in each group. ** *p* < 0.01 and **** *p* < 0.0001 compared with the control.

To further confirm the existence of apoA-I in apoA-I-containing nanoparticles (i.e., LT-rHDL, GM3-rHDL, and LT-GM3-rHDL) and to verify the apoA-I-mediated macrophage-targeting ability of these nanoparticles, an in vitro experiment via another microsphere-based method was performed (Figure 3). Only apoA-I-containing nanoparticles were able to link onto the surface of a streptavidin-coated microbead via the streptavidin-biotin and antibody (i.e., anti-apoA-I)-antigen (i.e., apoA-I) interactions and could be recognized by the receptors for apoA-I (e.g., SR-B1) expressed on the surface of cells (e.g., macrophages); therefore, the binding of microbeads onto cell surfaces could indirectly verify the existence of apoA-I and the apoA-I-mediated macrophage-targeting ability of the nanoparticles (Figure 3A). Our data showed that microbeads were observed on cells only in the apoA-I-containing groups (i.e., the LT-rHDL, GM3-rHDL, and LT-GM3-rHDL groups; Figure 3D,F,H, respectively) instead of the apoA-I-absent groups (i.e., the control, LT-NLC, GM3-NLC, and LT-GM3-NLC groups; Figure 3B,C,E,G, respectively) and that ~80% cells had microbeads (Figure 3I) with an average number of 3–4 beads per cell (Figure 3J) in the apoA-I-containing groups. Therefore, our data further confirmed the presence of apoA-I in apoA-I-containing nanoparticles and verified the apoA-I-mediated macrophage-targeting ability of the apoA-I-containing nanoparticles.

**Figure 4:**
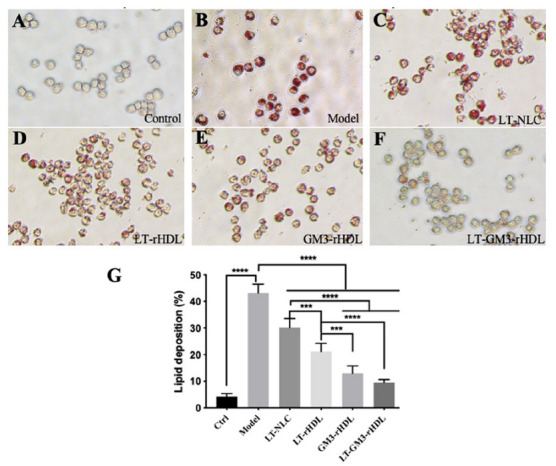
Comparison of LT-GM3-rHDL (or LT@GM3-rHDL) nanoparticles with other nanoparticles about the in vitro inhibitory effect on oxLDL-induced lipid deposition in macrophages. The RAW264.7 macrophages were incubated with the following samples for 24 h: medium only (i.e., control), oxLDL (100 μg/mL) only which was used to induce lipid deposition in cells (or foam cell formation), oxLDL + LT-NLC, oxLDL + LT-rHDL, oxLDL + GM3-rHDL, and oxLDL + LT-GM3-rHDL, respectively. After fixing with 2.5% glutaraldehyde, the cells were stained with Oil Red O. (**A**–**F**) Representative image in each group (magnification: 200×). (**G**) Quantification of lipid deposition (foam cell formation) in macrophages (*** *p* < 0.001 and **** *p* < 0.0001 compared with the indicated group).

In the intima of vessel wall, macrophage is the major cell type responsible for atherogenesis via the deposition of lipids (particularly cholesterol) derived from native or oxidized low-density lipoprotein (nLDL or oxLDL) under which condition macrophages are transformed into foam cells. To compare the effects of different nanoparticles on the lipid deposition in macrophages, as an in vitro atherosclerotic cell model cultivated macrophages were stimulated with 100 μg/mL oxLDL for 24 h to induce intracellular lipid deposition which were fluorescently stained in red with Oil Red O. Our data showed that a strong red fluorescence was observed in the model (oxLDL only) group, implying the successful induction of lipid deposition by oxLDL, and that other treatments (i.e., LT-NLC, LT-rHDL, GM3-rHDL, and LT-GM3-rHDL, respectively) caused decreases in fluorescence intensity to different extents (Figure 4A–F). The quantitative analysis also confirmed the observation and revealed that among these nanoparticles the LT-GM3-rHDL nanoparticle had the strongest in vitro inhibitory effect on oxLDL-induced lipid deposition (Figure 4G), implying the potentially higher antiatherosclerotic efficacy of the LT-GM3-rHDL nanoparticle than both the LT-rHDL and GM3-rHDL nanoparticles.

**Figure 5:**
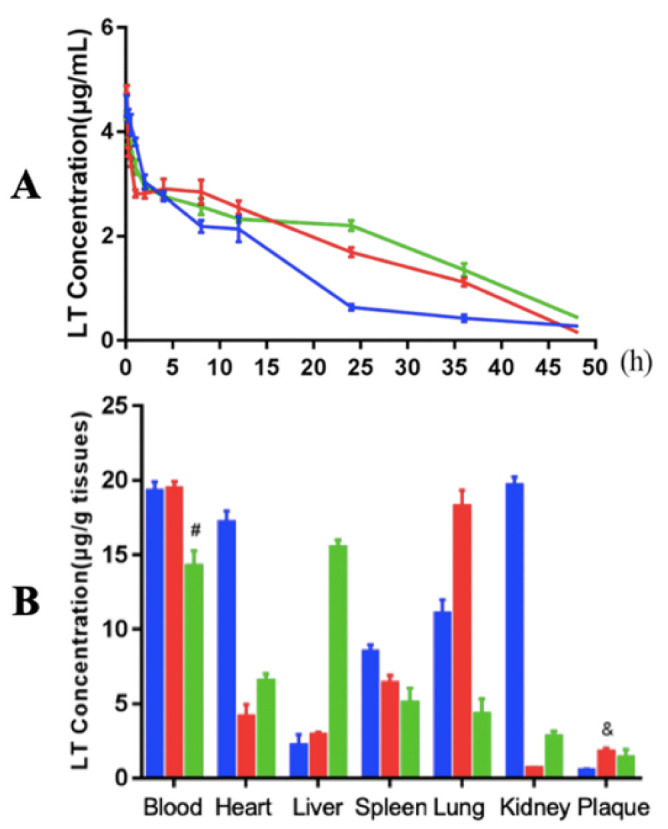
In vivo release and distribution of LT after a single i.v. drug administration of the indicated nanoparticles (i.e., LT-NLC in blue, LT-rHDL in red, and LT-GM3-rHDL in green, respectively). (**A**) Dynamic changes of LT concentration in the blood of atherosclerotic mice during 48 h after single administration. (**B**) The concentrations of LT in different tissues (blood, heart, liver, spleen, lung, kidney, and plaques, respectively) at 0.5 h after single administration (& and # represent *p* < 0.05 and *p* < 0.0001, respectively compared with LT-NLC).

Next, the in vivo drug (LT) release profiles in the blood of atherosclerotic mice during 48 hours after single administration of each of the nanoparticles (LT-NLC, LT-rHDL, and LT-GM3-rHDL nanoparticles, respectively) were determined. The data showed that the LT-rHDL and LT-GM3-rHDL nanoparticles have a more slowly sustained release of lovastatin than the LT-NLC nanoparticle (Figure 5A), coinciding with the in vitro drug release data (Figure 2E). Subsequently, the distributions of LT in different tissues (blood, heart, liver, spleen, lung, kidney, and plaques, respectively) at 0.5 h after single administration of each of these nanoparticles were detected (Figure 5B). Besides in blood, LT distributed predominantly in the heart, kidney, and lung for LT-NLC, whereas LT distributed mainly in the lung for LT-rHDL or in the liver for LT-GM3-rHDL. The atherosclerotic plaque in both LT-rHDL and LT-GM3-rHDL groups had a higher concentration of LT than that in the LT-NLC group, implying the plaque-targeting effect of apoA-I (or rHDL)-containing nanoparticles.

**Figure 6:**
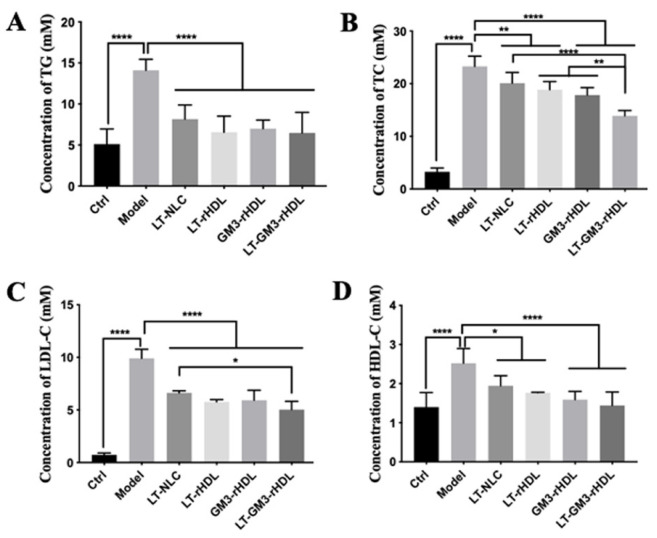
Comparison of LT-GM3-rHDL (or LT@GM3-rHDL) nanoparticles with other nanoparticles about the inhibitory effect on the concentrations of main blood lipids in apoE^−/−^ mice fed a high-fat diet for 12 weeks. The mice were categorized into 6 groups as follows: saline (control), model (high-fat diet), LT-NLC, LT-rHDL, GM3-rHDL, and LT-GM3-rHDL groups (in the last three groups the mice fed a high-fat diet were treated with the nanoparticles). (**A**) Total cholesterol (TG); (**B**) Triglyceride (TG); (**C**) LDL-cholesterol (LDL-C); (**D**) HDL-cholesterol (HDL-C). * *p* < 0.05, ** *p* < 0.01 and **** *p* < 0.0001 compared with the indicated group (n = 6).

**Figure 7:**
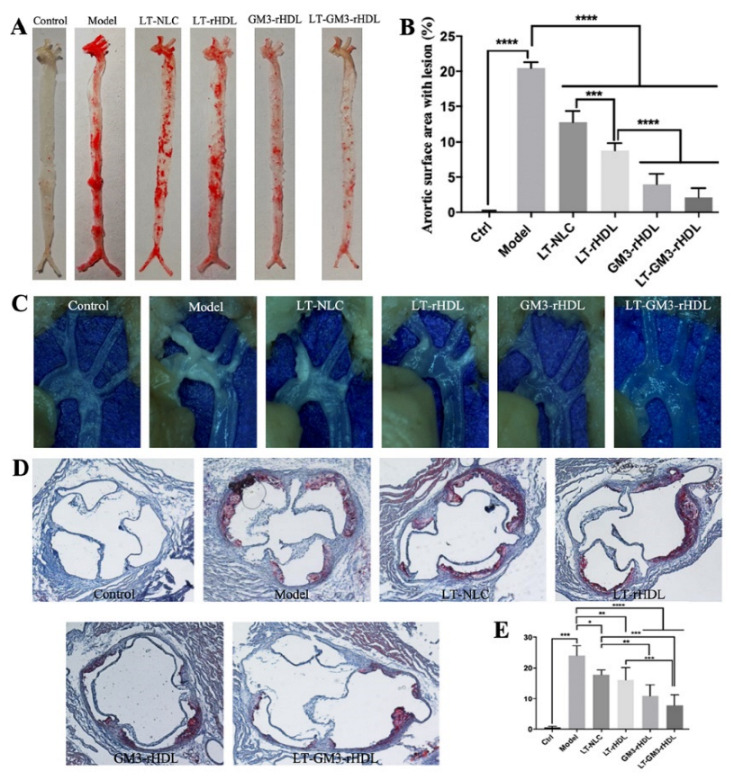
Comparison of LT-GM3-rHDL (or LT@GM3-rHDL) nanoparticles with other nanoparticles about the inhibitory effect on the formation of atherosclerotic lesions in apoE^−/−^ mice fed a high-fat diet for 12 weeks. (**A**) Representative images of full length aorta stained with Oil Red O staining. (**B**) Quantification of atherosclerotic plaques in full length aorta. (**C**) Representative images of aortic arches showing atherosclerotic plaques. (**D**) Representative images of aortic root slices stained with Oil Red O. (**E**) Quantification of atherosclerotic plaques in aortic root slices. * *p* < 0.05, ** *p* < 0.01, *** *p* < 0.001, and **** *p* < 0.0001 compared with the indicated group (n = 6).

Finally, to test the in vivo antiatherosclerotic effects of various nanoparticles, apoE-deficient (apoE^−/−^) mice fed a high-fat diet for 12 weeks were utilized to establish an atherosclerotic animal model. After treating with various nanoparticles during the period of 12 weeks, the lipid profiles (Figure 6) and the atherosclerotic plaques at different locations (Figure 7) were measured for evaluating the antiatherosclerotic effects of different nanoparticles. Compared with the control group (apoE^−/−^ mice fed a chow diet; the first panel of each graph in Figure 6 and Figure 7), the contents of blood lipids (including TG, TC, LDL-C, and HDL-C) and the areas of atherosclerotic plaques at various locations (i.e., aortic full length, aortic arch, and aortic root) dramatically increased in the model group (apoE^−/−^ mice fed a high-fat diet but without a drug treatment; the second panel of each graph in Figure 6 and Figure 7), confirming the successful establishment of the atherosclerotic mouse model. The experimental data also showed that compared with the model group all tested nanoparticles (i.e., LT-NLC, LT-rHDL, GM3-rHDL, and LT-GM3-rHDL nanoparticles, respectively) significantly decreased the contents of blood lipids and the areas of atherosclerotic plaques at various locations, confirming the lipid-lowering and antiatherosclerotic effects of LT and GM3 or GM3-rHDL, and that the LT-GM3-rHDL nanoparticle induced the most significant change (the last panel of each graph in Figure 6 and Figure 7), implying that the LT-GM3-rHDL nanoparticle indeed has the best in vivo antiatherosclerotic efficacy among the tested nanoparticles. However, the difference between GM3-rHDL and LT-GM3-rHDL was significant only for Figure 6B, probably due to the small study sample (i.e., type II error).

The better (although not significantly for some considered parameters in the vivo study) antiatherosclerotic efficacy of LT-GM3-rHDL than each of LT-rHDL, GM3-rHDL, and LT alone implies the possibility of GM3-containing rHDL (i.e., GM3-rHDL) as a novel nanocarrier (or an upgraded version of rHDL) of other traditional anti-atherosclerotic drugs (e.g., statins including lovastatin or LT), confirming our hypothesis mentioned in the Introduction section. As a drug nanocarrier, the efficacy-enhancing role of GM3-rHDL may benefit from the two players, rHDL and GM3, both of which have been previously reported to exert antiatherosclerotic efficacy (although a short-term beneficial effect for rHDL) by increasing the plaque targetability and/or cholesterol efflux for rHDL [26,29,30,31] and via multiple steps during atherogenesis for GM3 [13]. One possibility of the beneficial role of GM3 is that the addition of GM3 into rHDL nanoparticles may improve cellular uptake of the nanoparticles via the interaction between GM3 and CD169 (sialoadhesin or siglec-1) expressed on cells (e.g., T cells, dendritic cells, macrophages, etc.), based on which GM3-functionalized gold or polymer nanoparticles have been reported previously for viral research [32,33,34,35]. Another possibility is the lipid-lowering effect of GM3 [13]. The in vitro experimental data showed that GM3 could reduce the secretion of apolipoprotein B-100 (the major apolipoprotein in VLDL and LDL) in liver cells [36]. More in-depth studies will be needed to elucidate the underlying mechanisms. Out data imply that GM3-rHDL nanoparticle has the potential of being developed as a better delivery system for traditional antiatherosclerotic drugs (e.g., statins) than rHDL nanoparticle due to enhanced antiatherosclerotic efficacy by GM3.

## Figures and Tables

**Table 1:** Mean size, PDI, Zeta potential, EE, and DL (mean ± SD, n = 3).

	LT-NLC	LT-rHDL	GM3-rHDL	LT-GM3-rHDL
Mean size (nm)	86.3 ± 3.1	128.2 ± 1.5	114.8 ± 2.5	142.3 ± 3.6
Polydispersity index (PDI)	0.48 ± 0.03	0.35 ± 0.18	0.49 ± 0.03	0.46 ± 0.03
Zeta potential (mV)	−32.2 ± 2.4	−24.6 ± 0.6	−30.2 ± 3.2	−34.7 ± 3.4

## Data Availability

All data generated or analyzed during this study are included in this article.
